# Serum amyloid P ameliorates radiation-induced oral mucositis and fibrosis

**DOI:** 10.1186/1755-1536-3-11

**Published:** 2010-07-05

**Authors:** Lynne A Murray, Michael S Kramer, David P Hesson, Brynmor A Watkins, Edward G Fey, Rochelle L Argentieri, Furquan Shaheen, Darryl A Knight, Stephen T Sonis

**Affiliations:** 1 Promedior, Inc, 371 Phoenixville Pike, Malvern, PA, 19355, USA; 2 Biomodels and Affiliates, 313 Pleasant Street, Watertown, MA, 02472, USA; 3 Department of Anesthesiology, Pharmacology and Therapeutics, University of British Columbia, Burrard Street, Vancouver, Canada; 4 Department of Oral Medicine, Infection and Immunity, Harvard School of Dental Medicine Diviosn of Oral Medicine, Dana Farber Cancer Institute, Brigham And Women's Hospital, 75 Francis Street, Boston, MA, 02115, USA

## Abstract

**Purpose:**

To evaluate the effect of the anti-fibrotic protein serum amyloid P (SAP) on radiation-induced oral mucositis (OM) and fibrosis in a hamster cheek-pouch model.

**Experimental Design:**

Hamsters received a single dose of radiation (40 Gy) to the left everted cheek pouch to induce significant OM. The protective therapeutic potential of SAP was evaluated using varying dosing regimens. The extent of OM was measured using a validated six-point scoring scheme ranging from 0 (normal tissue, no mucositis) to 5 (complete ulceration). Fibrotic remodeling was also visualized histologically and quantified at later time points using collagen gene expression.

**Results:**

SAP treatment attenuated the profile of radiation-induced oral mucositis by delaying the time of onset, reducing the peak value, and enhancing the resolution of injury. The peak mucositis score was reduced by approximately 0.5 grade in SAP-treated animals. The number of animal days with a score of ≥ 3 was reduced by 48% in the SAP-treated group, compared with the saline control group (*P *< 0.01). SAP also inhibited the extent of tissue remodeling and decreased radiation-induced increases in myofibroblast number. Attenuated collagen deposition and gene expression was also observed in the cheek pouches of hamsters treated with SAP at both 16 and 28 days post-radiation.

**Conclusions:**

SAP treatment significantly attenuated radiation-induced injury. In particular, SAP attenuated the severity of OM and inhibited pathogenic remodeling. This suggests that SAP may be a useful therapy for the palliation of side effects observed during treatment for head and neck cancer.

## Background

Oral mucositis is a common side effect of chemotherapy and radiotherapy, with mucositis occurring to some degree in more than one-third of patients receiving anti-neoplastic drug therapy [1]. Moderate to severe mucositis occurs in virtually all patients who receive radiation therapy for tumors of the head and neck. It typically begins at cumulative exposures of 15 Gy and then worsens when total doses exceed 60 Gy [1-4]. The ensuing injury significantly impairs quality of life and can hamper the scheduled course of therapy, thus reducing efficacy of treatment. The complex pathoetiology is associated with the induction of a series of biologic pathways within the submucosa. Pronounced epithelial cell apoptosis and the generation of pro-inflammatory cytokines such as tumor necrosis factor (TNF)-α and interleukin (IL)-1β [5], and pro-fibrotic mediators such as transforming growth factor (TGF)-β [5], result in the breakdown of the oral mucosa, causing formation of ulcerative lesions. In patients with granulocytopenia, the ulcerations that accompany mucositis are common portals of entry for indigenous oral bacteria, often leading to sepsis or bacteremia [3]. Fibrotic remodeling of the damaged tissue then serves to seal off the region; however, if the extent of remodeling is overexuberant, the surrounding tissue becomes involved, resulting in a loss of elasticity that produces detrimental functional consequences. This, along with pronounced mucositis after directed radiotherapy or certain chemotherapies, can cause the patients to interrupt their scheduled treatments, thereby affecting long-term survival [3].

Serum amyloid P (SAP) is a member of the pentraxin family of serum proteins and has been previously shown to reduce fibrosis in experimental models [6-9]. This protein is highly conserved between species. *In vitro*, SAP has been described as inhibiting monocyte to fibrocyte differentiation and macrophage activation [9,10]. Fibrocytes are bone marrow-derived CD45+ collagen I+ cells that are thought to contribute to excess extracellular matrix (ECM) deposition at sites of fibrosis. *In vivo*, SAP also decreases fibrocyte and macrophage numbers in models of cardiac and pulmonary fibrosis [6,7]. More recently, an association of fibrocytes with both the prognosis and severity of interstitial lung disease has been described [11]. SAP also decreases the expression of macrophage activation markers in models of pulmonary and renal fibrosis [8,9].

The standard therapy for mucositis is predominantly palliative, including application of topical analgesics such as lidocaine and/or systemic administration of narcotics and antibiotics. In this study, we used an established hamster cheek-pouch model of radiation-induced mucositis to determine if SAP treatment affects either the clinical or pathological hallmarks of the disease. We further assessed the potential effect of SAP on the downstream fibrosis mediated by radiation.

## Methods

### Animals

Male Syrian golden hamsters (CRL) weighing approximately 80-90 g (5-6 weeks old, n = 12 per group) were used in this study. All experiments were conducted under Biomodels Institutional Animal Care and Use Committee regulations and protocols.

### Acute model of radiation-induced mucositis

Animals were given an acute radiation dose of 40 Gy directed to their left buccal cheek pouch, with the right cheek pouch serving as the non-irradiated control, as previously described [12]. Briefly, radiation was generated with a 160 KV potential (18.75 mA) source at a focal distance of 210 mm, hardened with a 3.0 mm Al filtration system (Kimtron Polaris II, Kimtron Inc, Woodbury, CT, USA). Irradiation targeted the left buccal pouch mucosa at a rate of 1.32 Gy/minute. Before irradiation, animals were anesthetized with intraperitoneal (i.p.) ketamine:xylazine (160 mg/kg:8 mg/kg). The left buccal pouch was everted and fixed, and isolated using a lead shield.

### SAP administration

SAP in 10 mM Tris, 140 mM NaCl buffer (EMD Biosciences, San Diego, CA, USA) was administered by i.p. injection at a dose of 2 mg/kg with dosing initiated 1 day before radiation (day -1) or immediately after radiation (day 0). Animals were dosed daily on days 0 to 7, every 2 days on days 0 to 12, or every 2 days on days -1 to 26. Additional groups of animals were dosed every 2 days with phosphate-buffered saline (PBS) on days 0 to 12 or days -1 to 26, and served as vehicle control groups. The dose level of 2 mg/kg SAP was chosen as this approximately doubles predicted endogenous SAP levels in the hamster.

### Evaluation of oral mucositis

Oral mucositis was evaluated from photographs by two independent, trained observers who were blinded to the study groups. The extent of mucositis was scored visually by comparison with a validated photographic scale (clinical scoring) (0 = completely healthy pouch with no vasodilation or erythema; 1 = light to severe erythema and vasodilation with no visible erosion of mucosa; 2 = severe erythema, vasodilation and erosion of the superficial mucosa; 3 = severe erythema and vasodilation, formation of off-white ulcers in ≥ 1 places with the cumulative size of the ulcers equaling approximately one-quarter of the pouch; 4 = severe erythema and vasodilation, cumulative size of ulcers encompassing approximately half the pouch; 5 = virtually all of the pouch is ulcerated, loss of pliability with the pouch only partially extractable from the mouth).

### Gene expression analysis

For gene analysis, cheek pouch tissue was homogenized in lysis buffer (Panomics, Freemont, CA, USA), in accordance with the manufacturer's instructions. Owing to the high levels of collagen present in the hamster cheek pouch, homogenized samples were passed through a 0.45 μm cellulose nitrate plate (Whatman, Piscataway, NJ, USA) before mRNA analysis. mRNA levels were quantified using a branched-DNA technology-based method (QuantiGene Reagent System; Panomics), according to the manufacturer's protocols. Transcript levels of fibrosis and macrophage-related genes were normalized to β-actin mRNA.

### Histology

Cheek pouches fixed in 10% normal buffered formalin (NBF) were cut into horizontal sections perpendicular to the long axis of the cheek pouch. Samples were changed to 70% alcohol, embedded in paraffin and cut into sections approximately 5 μm thick. Serial sections were stained with hematoxylin and eosin (H&E) for gross morphology, or Masson's trichrome stain for collagen deposition. Sections were examined using am inverted microscope and camera (Micromaster; Fisher Scientific Pittsburgh, PA, USA).

#### Histological fibrosis scoring

H&E-stained slides were assessed for fibrosis according to a six-point scale (0 = no fibrosis noted; 1 = minimal fibrosis; 2 = mild fibrosis; 3 = moderate fibrosis; 4 = marked fibrosis; 5 = severe fibrosis). Analysis was directed by a board-certified veterinary pathologist blinded to the study group designation.

### Immunohistochemistry

Formalin-fixed, paraffin wax-embedded cheek pouch sections were analyzed for immunohistochemical localization of α-smooth muscle actin (α-SMA) expression using an indirect immunoperoxidase procedure. Sections were dewaxed with xylene, rehydrated in graded concentrations of ethanol, and blocked with normal horse serum. Mouse anti-α-SMA monoclonal antibody (A5228, clone 1A4; Sigma-Aldrich, Missouri, MO, USA) and control normal rabbit IgG were diluted in PBS to a final concentration of 5 μg/ml. Anti-α-SMA or IgG were added to histological sections for 60 min, after which each tissue section was washed thoroughly with PBS. A secondary biotinylated donkey-anti-mouse IgG (Jackson ImmunoResearch, West Grove, PA, USA) was added to each section for 1 hour. Slides were then thoroughly washed and α-SMA visualized with a commercial staining kit (HRP-Dab Staining Kit; Vector Laboratories, Burlingame, CA, USA).

### *In vitro *fibroblast assay

Normal human dermal fibroblasts (NHDF) were plated into 24-well plates (Costar, Corning, NY, USA) at 100,000 cells/well, and allowed to adhere for 8 hours. The cells were then washed with PBS and cultured overnight in serum-free media (Dulbecco's modified Eagle's medium (DMEM) with L-glutamine, penicillin and streptomycin). Cells were then stimulated for 24 hrs in the presence or absence of platelet-derived growth factor (PDGF) antibody (200 ng/mL) and/or SAP (1 or 4 μg/mL). Proliferation was assessed a cell proliferation ELISA with 5-bromo-2-deoxyuridine incorporation (Roche Applied Science, Roche Diagnostic Corporation, Indianapolis, IN, USA).

### Epithelial to mesenchymal transition assay

Experiments were performed in A549 cells grown to 60% confluence in six-well plates (BD Biosciences, Mississauga, ON, USA), which were grown in DMEM containing 10% fetal bovine serum (FBS) at 37°C in 5% CO_2 _in air. Before each experiment, cells were incubated in DMEM with 0.5% FBS for 24 hours. A549 cells were then incubated with or without TGF-β_1 _(10 ng/mL) or SAP (1, 5 or 10 μg/mL) or both for 48 hours. After this, cells were lysed in protein extraction buffer supplemented with phenylmethanesulfonyl fluoride, phosphatase inhibitor cocktail 2, and protease inhibitor cocktail (Sigma-Aldrich). Lysates at a concentration of 50 ng/ml were then separated by electrophoresis in SDS-PAGE gels and electrotransferred to nitrocellulose membranes, which then underwent western blotting analysis with antibodies directed against extra domain-A (EDA)-fibronectin (EDA-FN) (MAB1940; Chemicon International, Temecula, CA, USA) and E-cadherin (SC-8426, Santa Cruz Biotechnology, Santa Cruz, CA, USA). Hsp90 (BD610418, BD Biosciences, Mississauga, ON, USA) was used as a protein loading control.

### Statistics

For each evaluation day, the scores of the vehicle control group were compared with those of the treated groups using non-parametric rank sum analysis. Statistical differences between treatment groups were determined using the Student *t*-test, Mann-Whitney *U *test and χ^2 ^analysis, with a critical value of 0.05 as appropriate. *P *values of <0.05, <0.01 or *P *< 0.005 *referred to as *, ** or ***, respectively) were considered significant.

## Results

### Radiation-induced fibrosis

**Figure 1:**
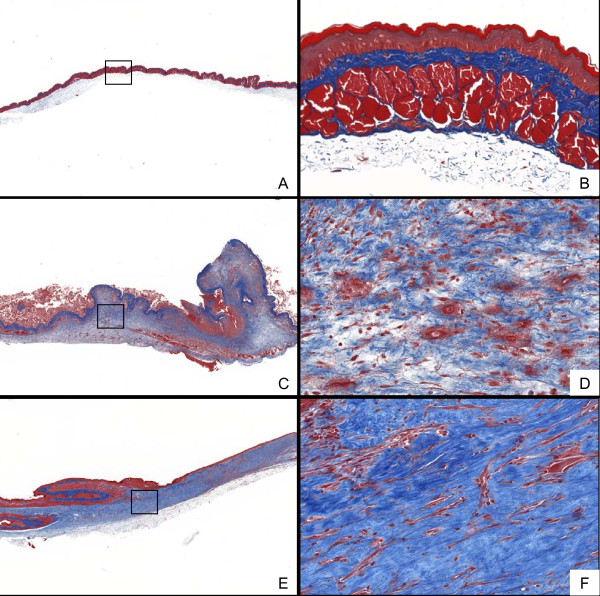
**Representative histopathology of radiation-induced fibrosis**. Hamsters were exposed to radiation (40 Gy) on day 0. At subsequent time points, cheek pouches were removed, sectioned and stained with Masson's trichrome to visualize collagen deposition. Representative cheek pouch sections are shown for **(A,B) **non-irradiated controls, **(C,D) **day 16 post-radiation, and **(E,F) **day 28 post-radiation.

The clinical course of mucositis is well characterized in this hamster cheek-pouch model of radiation-induced mucositis [12], with peak mucosal breakdown occurring between days 14 and 18. The development of ulcerative mucositis in this study was consistent with historical data from our laboratory. To assess the extent of downstream tissue remodeling as it pertains to collagen deposition, hamsters received a single dose of radiation (40 Gy) specifically to the left everted cheek pouch on day 0 and were killed 16 or 28 days later. Cheek pouches were sectioned and stained with Masson's trichrome to visualize areas of excess collagen accumulation (Figure 1). In non-irradiated cheek pouches, densitometric analysis showed approximately 43% trichrome positivity in each of the cheek pouches analyzed (Figure 1A, B). At day 16 in irradiated cheek pouches, there were large areas of ulceration densely infiltrated with neutrophils, with surrounding necrosis in the muscle layer (Figure 1D). Irregular regions of fibrosis were observed beneath the muscular layer of the buccal pouch (Figure 1C). At day 28, there were fewer regions of epithelial ulceration, and the extent of inflammation was also reduced, indicating a degree of mucositis resolution at the histopathological level (Figure 1F). However, the cheek pouches exhibited more pronounced fibrosis at day 28 (60% trichrome positivity; Figure 1E), with the aberrant ECM being better organized compared with the day 16 time point (58% trichrome positivity; Figure 1C). This increase in ECM deposition indicates that the hamster cheek-pouch model is suitable to assess the anti-fibrotic potential of SAP as it pertains to radiation-induced fibrosis.

### SAP inhibits radiation-induced mucositis

**Figure 2:**
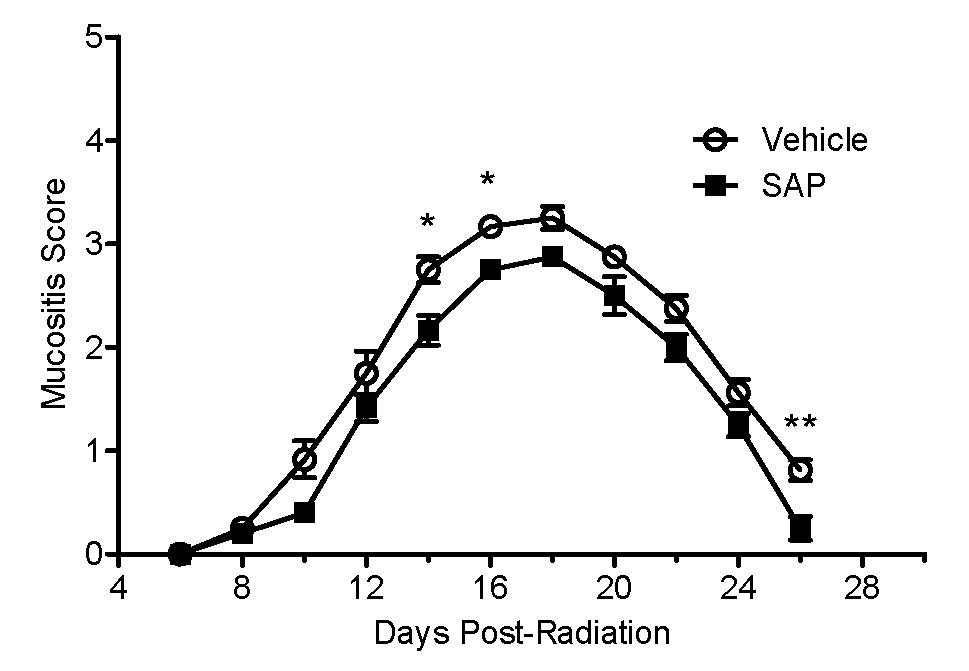
**Effect of serum amyloid P (SAP) on radiation-induced mucositis**. Hamsters were exposed to radiation and treated with SAP (2 mg/kg, i.p.) every 2 days from days 0 to 12. Mean mucositis scores were determined in phosphate buffered saline (vehicle)-treated (open symbols; n = 12) and SAP-treated (filled symbols; n = 12) animals from days 6 to 26. *P < 0.05, **P < 0.01 compared with PBS vehicle-treated control irradiated hamster cheek pouches.

The effect of SAP on radiation-induced mucositis was initially assessed by administering SAP (2 mg/kg, i.p.) to the hamsters every 2 days from days 0-12 after radiation exposure. In control (PBS-treated) animals, the peak in mucositis occurred at day 18, with a mean ± SEM mucositis score of 3.25 ± 0.11 (Figure 2). After the peak at day 18, the extent of mucositis subsided, with a mean mucositis score of 0.81 ± 0.10 on day 6. In the SAP-treated animals, the peak in radiation-induced mucositis also occurred at day 18. However, the magnitude of mucositis was lower (2.88 ± 0.09), with significantly reduced mucositis scores being observed in the SAP-treated (day 0-12) animals at days 14 and 16 compared with vehicle controls (*P *< 0.05). In fact, the SAP-treated animals never reached a group mean of 3 throughout the study, whereas vehicle-treated animals reached a group mean of >3 by day 16. Another significant effect of SAP was observed at day 26, with SAP-treated animals having a lower mean mucositis score (0.25 ± 0.11) than vehicle control hamsters (*P *< 0.01). Totaling the number of days that animals had a mucositis score of ≥ 3 (severe mucositis and of clinical significance), revealed that animals treated with SAP had a significantly attenuated duration of severe mucositis (48% relative reduction, *P *< 0.01). Taken together, these results suggest that SAP treatment delays the onset, reduces the peak dampens the total extent, and promotes resolution of mucositis.

### SAP inhibits radiation-induced fibrosis in the hamster cheek pouch

**Figure 3:**
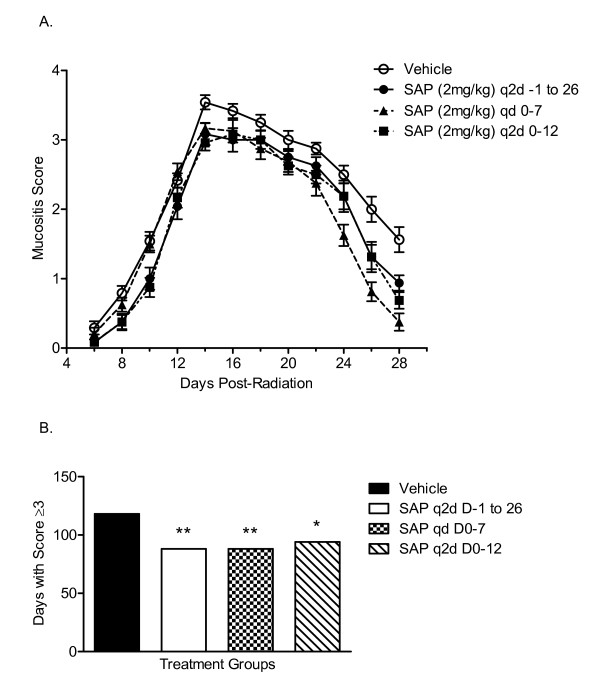
**Attenuation of radiation-induced mucositis with an altered serum amyloid P (SAP) dosing schedule**. Hamsters were exposed to radiation and treated with SAP (2 mg/kg, i.p. every 2 days from day 0 to 12, daily from days 0 to 7, or every 2 days from days -1 to 26 (n = 12 per group) or vehicle control (phosphate-buffered saline (PBS), i.p. every 2 days from days 0 to 12; n = 12). **(A) **Mean mucositis scores were determined in PBS (vehicle)-treated (open symbols) and SAP-treated (filled symbols) animals from days 6 to 28. **(B) **The total number of days that hamsters had a mucositis score of ≥ 3 was calculated. *P < 0.05, **P < 0.01 compared with PBS vehicle-treated control irradiated hamster cheek pouches.

**Figure 4:**
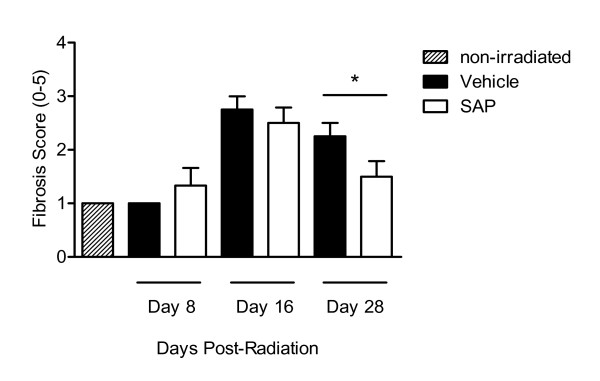
**Reduction in radiation-induced fibrosis mediated by serum amyloid P (SAP)**. Hamsters were exposed to radiation (40 Gy) on day 0. At subsequent time points, cheek pouches were removed, sectioned and stained with haematoxylin and eosin, and extent of fibrosis was assessed. Original magnification: left, × 11; right × 200 (region in the inset box). Bars represent the mean ± SEM of four animals per group.

Histological analysis and quantification of H&E-stained sections of the cheek pouches of hamsters exposed to radiation, assessing both fibrotic changes and overt inflammation, indicated that there was a time-dependent increase in overall pathogenic changes, with scores peaking at day 16 (2.75 ± 0.25) and showing only a moderate reduction by day 28 (2.25 ± 0.25) (Figure 3). As with vehicle controls, hamsters treated with SAP (day 0-12) had increased fibrous material at day 16 (2.50 ± 0.29) compared with non-irradiated control cheek pouches and compared with day 8 hamster cheek pouches. However, there was significantly reduced fibrous material in the cheek pouches of animals treated with SAP at day 28 (1.50 ± 0.29) compared with vehicle control-treated animals (*P *< 0.05; Figure 4).

### Acute dosing with SAP at the time of radiation gives greatest protection

To determine the optimum dosing strategy of SAP to attenuate radiation-induced mucositis and fibrosis, we assessed the efficacy of various dosing schedules. Hamsters were treated with SAP immediately after radiation (day 0) and given SAP either every 2 days from day 0 to 12 or daily from days 0 to 7. In another group of animals, SAP treatment was initiated 1 day before radiation and was given every 2 days until the end of the study (days -1 to 26). Mucositis scoring indicated that all dosing regimens reduced the peak of mucositis and enhanced its resolution (Figure 3A), thus attenuating the overall time that animals actually had severe mucositis (Figure 3B).

**Figure 5:**
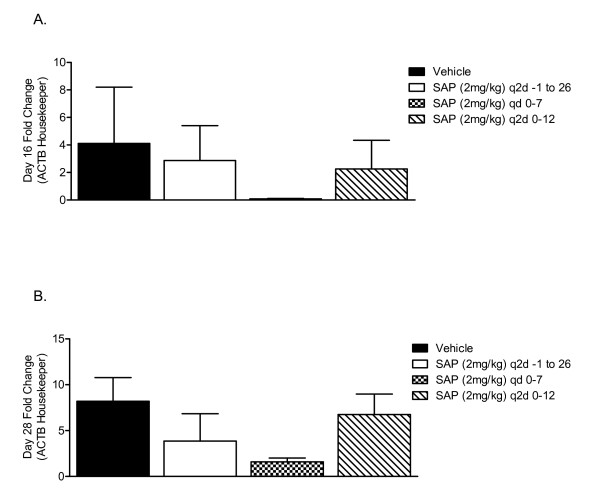
**Attenuated fibrosis-associated gene expression after serum amyloid P (SAP) treatment**. Hamsters were exposed to radiation and treated with SAP (2 mg/kg, i.p. every 2 days from days 0 to 12, daily from days 0 to 7, or every 2 days from days -1 to 26) or vehicle control (phosphate-buffered saline (PBS), i.p. every 2 days from days 0-12). Procollagen III gene expression was determined in the irradiated cheek pouches using branched DNA technology at **(A) **day 16 and **(B) **day 28. Bars represent the mean ± SEM. of four animals per group.

**Figure 6:**
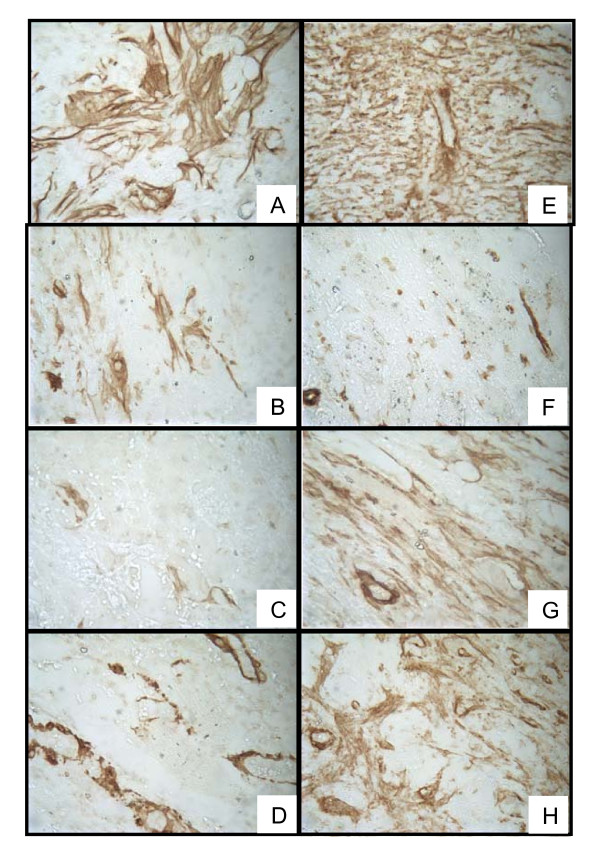
**Reduction in radiation-induced α-smooth muscle actin (α-SMA) expression with serum amyloid P (SAP) treatment**. Hamsters were exposed to radiation and treated with SAP (2 mg/kg, i.p. every 2 days from days 0 to 12, daily from days 0 to 7, or every 2 days from days -1 to 26) or vehicle control (phosphate-buffered saline (PBS), i.p. every 2 days from days 0 to 12). At either 16 or 28 days post-radiation, animals were killed and cheek pouches removed, sectioned and stained with anti-α-SMA to visualize myofibroblast accumulation and vascular smooth muscle cells. **(A-H) **Representative histopathology from irradiated cheek pouches from the vehicle groups **(A) **at day 16 and **(B) **day 28; from the first SAP group (2 mg/kg every 2 days, days -1 to 26) at **(C) **day 16 and **(D) **day 28; from the second SAP group (2 mg/kg daily, days 0 to 7) at **(E) **day 16 and **(F) **day 28; and from the third SAP group (2 mg/kg every 2 days, days 0 to 12) at day 16 **(G) **and day 28 **(H)**.

**Figure 7:**
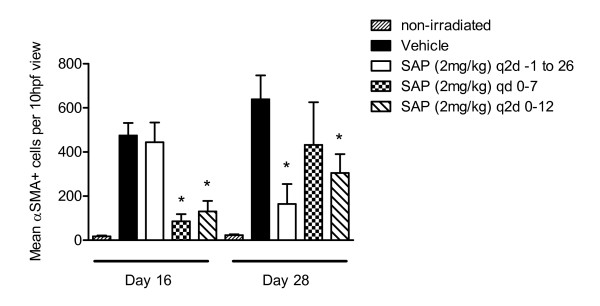
**The total number of α-smooth muscle actin (α-SMA)-positive cells was quantified morphometrically. Bars represent the mean ± SEM of four animals per group**. *P < 0.05 compared with PBS (vehicle) control irradiated hamster cheek pouches. Original magnification × 400.

Analysis of procollagen III gene expression in the hamster cheek pouches, as a surrogate for the extent of fibrosis, determined that hamsters treated with SAP daily from days 0-7 had significantly less procollagen III gene transcript compared with the PBS vehicle-treated or the hamsters treated with the other two SAP dosing strategies when measured at day 16 (Figure 5A) or day 28 (Figure 5B). Quantification of myofibroblast number, as visualized by immunohistochemical staining of α-SMA (Figure 6A-H) confirmed the anti-fibrotic activity of SAP at day 16 in animals dosed on days 0-7 (Figure 7). However there were also significantly fewer α-SMA positive cells at both day 16 and day 28 in animals dosed every 2 days from days 0 - 12, and also at day 28 in the animals dosed with SAP throughout the study (Figure 7).

### SAP does not promote fibroblast proliferation or epithelial to mesenchymal transition

**Figure 8:**
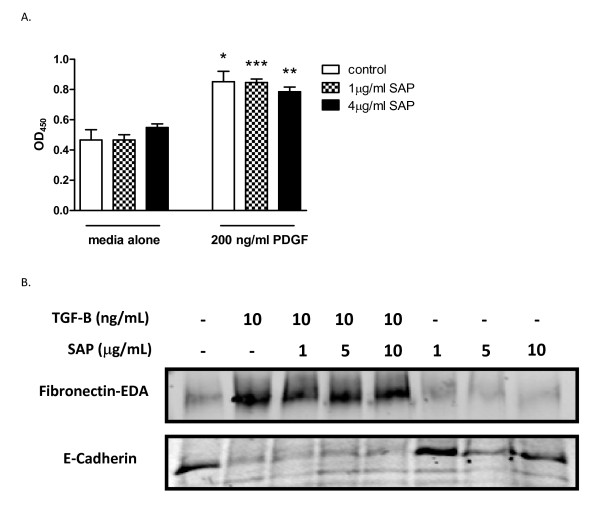
**Serum amyloid P (SAP) has no effect on fibroblast proliferation or transforming growth factor (TGF)-β-mediated epithelial to mesenchymal transition (EMT)**. (A) Normal human dermal fibroblasts (NHDF) were stimulated with platelet-derived growth factor (PDGF; 200 ng/mL) for 24 hours in the presence or absence of SAP. Proliferation was quantified using 5-bromo-2-deoxyuridine incorporation. **(B) **A549 cells were stimulated with TGF-β1 in the presence or absence of SAP for 48 hours. The resultant lysates were assessed for the epithelial marker E-cadherin or the mesenchymal marker extra domain-A (EDA)-fibronectin using western blotting analysis. *P < 0.05, **P < 0.01, ***P < 0.005.

To determine the potential mechanistic anti-fibrotic activity of SAP on mesenchymal cells, we initially assessed the effect(s) of SAP on fibroblast proliferation, alone or in combination with known proliferative mediators. As expected, PDGF promoted significant proliferation (Figure 8). By contrast, SAP had no direct proliferative effect, nor did it modulate PDGF-induced proliferation when added concomitantly (Figure 8A). We also assessed the effects of SAP on epithelial to mesenchymal transition (EMT) using the human lung epithelial cell line A549. Incubation with TGF-β1 at 10 ng/ml induced phenotypic changes consistent with EMT (Figure 8B). Morphologically, the cells lost their rounded cobblestone appearance and became stellate. Exposure to TGF-β1 also induced expression of the mesenchymal marker EDA-FN, together with downregulation of the epithelial marker E-cadherin (Figure 8B). Addition of SAP (1-10 μg/ml) alone had no obvious effect on the phenotype of A549 cells or on the expression of E-cadherin or EDA-FN (Figure 8B). Similarly, when added at the same time as TGFβ1, SAP had no effect on TGFβ1-induced changes in cell morphology or on expression of EDA-FN or E-cadherin (Figure 8B).

## Discussion

We have shown that SAP treatment results in a significant reduction in the two pathological sequelae of clinical radiotherapy and chemotherapy, mucositis and fibrosis. In the oral mucosa, directed radiation therapy induces apoptosis of the epithelial barrier which initiates an inflammatory cascade, characterized by nuclear factor (NF)κB activation [13]. The ensuing fibrotic response serves to provide adequate local wound healing; however, this can be overexuberant and limit tissue elasticity and function. SAP treatment dampened the extent of mucositis, as demonstrated by a reduction in the time to onset and attenuation in both the peak and duration of pathology. SAP also attenuated the fibrotic wound healing response.

In the present study, oral mucositis was scored using the established WHO criteria [14]. Using this scale, a score of ≥ 3 [represents severe mucositis that affects a patient's quality of life, with 70% of patients requiring feeding tubes to maintain adequate nutrition [14]. Further, in approximately 35% of patients with grade 3-4 mucositis, subsequent rounds of radiotherapy may be delayed or prevented, leading to less successful clinical therapy. Lower grades of mucositis may also significantly affect patient quality of life. Grade 1 is associated with pain that is sufficiently severe to require opioid analgesia. At grade 2, patients have difficulty or an inability to swallow due to ulcerations in the mouth and throat, which, if severe, may necessitate total parenteral nutrition and rehydration. Early expression of mitogen-activated protein kinase (MAPK) is observed within 8 hours post-radiation [15]. MAPK may function with NF-κB as a coactivator of a large number of genes involved in mucosal injury [16]. The activation of NF-κB due to radiation results in upregulation of a variety of genes leading to increased production of pro-inflammatory cytokines including TNF-α, IL-1β and IL-6 [17]. Targeted radiation has been shown to increase cyclooxygenase-2 expression in submucosal tissues, and this parallels the development of ulcerative mucositis [18]. In this study, we show that SAP treatment reduced the number of days that animals had a mucositis score of ≥ 3, indicating that SAP may provide significant palliative care, allowing the continuation of radiotherapy. Histopathologic assessment of cheek pouches indicated a significant reduction in the extent of leukocyte infiltration after SAP treatment. SAP has recently been shown to reduce inflammation in several experimental models of fibrosis through an Fc receptor-dependent mechanism [7,9,19]. Therefore the anti-inflammatory effect of SAP treatment on mucositis may occur via a similar mechanism. Owing to the model limitations, with limited hamster reagents available, we were confined by the number of endpoints we could assess.

The pathogenesis of mucositis is multifactorial, involving the interaction of oral mucosal epithelial cells, endothelial cells, connective tissue and the submucosal infiltrate [5]. There are four interdependent phases: (1) an inflammatory/vascular phase, (2) an epithelial phase, (3) an ulcerative/bacteriological phase, and finally (4) a healing/resolution phase [20]. Radiation directly damages cellular DNA, which results in cellular apoptosis and also inhibits homeostatic cell renewal. The debris generated during apoptosis promotes inflammation via numerous cascades including Toll-like receptor signaling and NFκB activation. Non-phlogistic clearance of these pro-inflammatory signals by macrophages acts to switch off the immune response, thus limiting damage to host tissue [21]. Inadequate clearance by macrophages has been shown to contribute directly to a number of acute and chronic diseases [22]. SAP clears apoptotic and necrotic debris [9,23], which then helps to promote a quiescent clearance of the damage-associated molecular patterns (DAMPs), thus minimizing the extent of inflammation. Further, inhibiting apoptosis has been shown to reduce oral mucositis [24]. Subsequent studies assessing the effect(s) of SAP on radiation-induced apoptosis and necrosis would help to determine if this is part of the protective therapeutic mechanism.

The ensuing fibrotic response mediated by radiation is associated with an increase in ECM deposition. Fibroblasts, when activated, produce various ECM components and can also differentiate into myofibroblasts, which are more contractile and more readily synthesize ECM, resulting in a greater loss of tissue elasticity. SAP decreased the amount of ECM and the number of myofibroblasts in the cheek pouch after radiation. SAP did not directly modulate fibroblast proliferation; therefore this anti-fibrotic mechanism *in vivo *is upstream of direct fibroblast activity.

Another key stromal cell type demonstrated to promote fibrosis is the epithelial cell. Epithelial cells stimulated with the prototypic growth factor TGF-β result in a transition of cell phenotype away from the resident epithelial cell and towards a motile, ECM-producing mesenchymal cell. This process is referred to as epithelial to mesenchymal transition (EM)T and is understood to contribute to lung, liver and kidney fibrosis [25-27]. TGF-β is the prototypic inducer of EMT. However, we determined that SAP did not modulate TGFβ-induced EMT, nor did SAP have any direct EMT effects.

Recent studies have indicated that during fibrosis, SAP directs monocyte to profibrotic fibrocyte or M2 macrophage differentiation [8-10]. This suggests that the reduction in myofibroblast number in the cheek pouch after SAP treatment is due to a mechanism upstream of direct modulation of fibroblast activity and may be due to modulation of monocyte differentiation. Future studies assessing the monocyte/macrophage phenotype in the cheek pouch should determine if there is also a profibrotic phenotype associated with the pathology observed in this model, as has been reported clinically [28]. Further, determining whether SAP can direct the macrophage phenotype away from a fibrotic M2 phenotype and towards a classic M1 macrophage phenotype will be insightful.

The observed utility of SAP as a therapeutic for mucositis in reducing both the acute inflammation and the downstream fibrosis is exciting. Various potential therapeutics have been assessed clinically for oral mucositis including recombinant IL-11 [29], granulocyte-macrophage colony-stimulating factor (GM-CSF) [30,31], G-CSF [32] and TGF-β3 [33]. Currently, the only approved treatment for mucositis is palifermin (Kepivance^®^; Biovitrum, Stockholm, Sweden), which is approved for the treatment of oral mucositis in patients undergoing bone marrow ablation for transplant. Palifermin is a recombinant keratinocyte growth factor and, as with all the other agents tested clinically, promotes epithelial cell proliferation, thus providing a denser barrier to protect the underlying mucosal tissue. By contrast, we have demonstrated that SAP has no direct pro-proliferative response on resident cells, nor does it directly inhibit epithelial cell transition during fibrosis. Importantly, SAP has more profound effects on the downstream responses mediated by radiotherapy, namely reducing the ensuing inflammation and thus limiting stromal cell activation, as demonstrated by reduced ECM deposition and α-SMA expression *in vivo*.

## Competing interests

Promedior sponsored this study. Promedior are developing SAP for the treatment of fibrotic diseases. LAM, MSK, DPH and RLA were employed by Promedior during this study.

## Authors' contributions

LAM conceived the study, participated in design and coordination, and drafted the manuscript. MSK, DPH, STS conceived the study, and participated in design and coordination. BAW, EGF, RLA and FS participated in design and coordination, and conducted the studies. DAK participated in design and coordination. All authors read and approved the final manuscript.
